# HIV-1 Mutation and Recombination Rates Are Different in Macrophages and T-cells

**DOI:** 10.3390/v8040118

**Published:** 2016-04-22

**Authors:** Deborah Cromer, Timothy E. Schlub, Redmond P. Smyth, Andrew J. Grimm, Abha Chopra, Simon Mallal, Miles P. Davenport, Johnson Mak

**Affiliations:** 1 Infection Analytics Program, Kirby Institute, UNSW Australia, Sydney NSW 2052, Australia; d.cromer@unsw.edu.au (D.C.); andrew.j.grimm@gmail.com (A.J.G.); 2 Centre for Vascular Research, UNSW Australia, Sydney NSW 2052, Australia; 3 Sydney School of Public Health, Sydney Medical School, University of Sydney, Sydney NSW 2006, Australia; tim.schlub@sydney.edu.au; 4 Centre for Virology, Burnet Institute, Melbourne VIC 3004, Australia; r.smyth@ibmc-cnrs.unistra.fr; 5 Architecture et Réactivité de l’ARN, IBMC, CNRS, Université de Strasbourg, 67084 Strasbourg, France; 6 Institute for Immunology and Infectious Diseases (IIID), Murdoch University, Perth WA 6150, Australia; a.chopra@iiid.com.au (A.C.); S.Mallal@iiid.com.au (S.M.); 7 Biosecurity Flagship, CSIRO (AAHL), Geelong VIC 3220, Australia; 8 School of Medicine, Deakin University and CSIRO (AAHL), Geelong VIC 3216, Australia

**Keywords:** HIV, mutation, recombination, evolution

## Abstract

High rates of mutation and recombination help human immunodeficiency virus (HIV) to evade the immune system and develop resistance to antiretroviral therapy. Macrophages and T-cells are the natural target cells of HIV-1 infection. A consensus has not been reached as to whether HIV replication results in differential recombination between primary T-cells and macrophages. Here, we used HIV with silent mutation markers along with next generation sequencing to compare the mutation and the recombination rates of HIV directly in T lymphocytes and macrophages. We observed a more than four-fold higher recombination rate of HIV in macrophages compared to T-cells (*p* < 0.001) and demonstrated that this difference is not due to different reliance on C-X-C chemokine receptor type 4 (CXCR4) and C-C chemokine receptor type 5 (CCR5) co-receptors between T-cells and macrophages. We also found that the pattern of recombination across the HIV genome (hot and cold spots) remains constant between T-cells and macrophages despite a three-fold increase in the overall recombination rate. This indicates that the difference in rates is a general feature of HIV DNA synthesis during macrophage infection. In contrast to HIV recombination, we found that T-cells have a 30% higher mutation rate than macrophages (*p* < 0.001) and that the mutational profile is similar between these cell types. Unexpectedly, we found no association between mutation and recombination in macrophages, in contrast to T-cells. Our data highlights some of the fundamental difference of HIV recombination and mutation amongst these two major target cells of infection. Understanding these differences will provide invaluable insights toward HIV evolution and how the virus evades immune surveillance and anti-retroviral therapeutics.

## 1. Introduction

One of the hallmarks of human immunodeficiency virus (HIV) is the high level of genetic diversity that can be observed within a single patient as a result of mutation and recombination of the HIV genome. This variation presents a “moving target” for the immune system, allowing HIV to escape immune recognition and persist within an infected individual. Additionally, rapid viral evolution allows HIV to develop resistance to antiretroviral therapy and so limits the effectiveness of drug regimes. The viral diversity and evolution of HIV is a product of high viral turnover [1], mutation and recombination during reverse transcription [2], and selection from the immune system [3,4]. Mutation rates are driven by the error prone reverse transcriptase enzyme, which introduces mutations at a level of 0.1 to 1 per genome, per replication cycle [5,6,7,8]. Additional genetic diversity can also be introduced during reverse transcription when the reverse transcriptase enzyme switches between two co-packaged RNA strands, incorporating part of the sequence from each strand into the resulting complementary DNA (cDNA). When these two strands are non-identical, this process of template switching is referred to as recombination and results in a new, shuffled genetic variant.

Whilst a large number of studies have measured mutation and recombination rates in cell lines [5,6,7,9,10,11,12], there have been comparatively few studies using primary macrophages and T-cells, the natural target cells of HIV-1 infection [8,13,14,15,16]. Moreover, recombination rate measurements using primary T-cells and macrophages have led to conflicting results, with some reports suggesting that recombination in macrophages may be higher than the rate in T-cells; however this remains controversial [13,14,15]. Surprisingly, given the importance of mutation to the evolution of drug resistance and immune escape, there have been no direct comparisons of the HIV-1 mutation rate between primary T-cells and macrophages that have been based on a primary sequencing approach.

Mutation and recombination rates can be directly and indirectly modulated by host cellular factors [2]. Cellular nucleic acid editing enzymes, exemplified by the apolipoprotein B mRNA-editing enzyme catalytic polypeptide-like 3 (APOBEC3) protein family, can directly introduce large numbers of mutations into the viral cDNA [17]. On the other hand, both recombination and mutation can be indirectly modulated by cellular factors such as deoxynucleoside triphosphate (dNTP) levels, which are, in turn, influenced by a myriad of factors, such as the cell type, activation status and the presence of certain viral and cellular proteins [9,15]. Previous studies comparing the recombination rate in T-cells and macrophages have used a non-viral gene reporter system, rather than measuring this rate directly from DNA sequencing [13,14,15]. Moreover, some of these reporter systems lack viral accessory genes that may indirectly influence the overall rate of genetic diversification [13,15]. Therefore we sought to compare the average rate of recombination during macrophage or T cell infection, as well as the positions of hot and cold spots for recombination in these two primary cells that are naturally infected by HIV. We have modified a full-length HIV provirus enabling us to simultaneously and directly measure mutation and recombination. We have previously used this system to show that HIV-1 infection of T-cells results in an average of 12 to 14 template switches per replication cycle, allowing multiple opportunities for the generation of viral diversity [8,16,18]. Furthermore, we have previously shown that 15%–20% of substitution mutations are associated with recombination in T-cells [8]. Here, we directly compare mutation and recombination rate between T-cells and macrophages, finding a higher recombination rate (6.18/1000nt *vs.* 1.46/1000nt) in macrophages compared to T-cells respectively, but interestingly a lower mutation rate (0.091/1000nt *vs.* 0.12/1000nt respectively). Although we found similar recombination hotspots between the two cell types, we were unable to observe an association between mutation and recombination in macrophages.

## 2. Materials and Methods

### 2.1. Molecular Clones

The HIV-1 molecular clones have been previously described [8,16,18]. Briefly, the wild-type (WT) molecular clone pDRNLAD8 is based on the prototypic HIV strain pNL43, engineered to remove 1.5 kb of cellular DNA flanking the HIV-1 genome in the pNL43 construct [19], and to express the R5-tropic AD8 envelope. The marker (MK) molecular clone pDRNLAD8MK_high_ is a modified pDRNLAD8 clone engineered to contain 15 and 34 silent marker points in *gag* and *pol*, respectively. This resulted in 47 different genome regions in which recombination could be studied. Marker points largely consisted of two single base pair changes in adjacent codons to enable recombination to be easily distinguished from mutation (full details can be found in [8,16,18]). Each region ranged between 17 and 155 (median 47) nucleotides in length. We also used a second marker clone, pDRNLAD8MK_low_ that contained 17 and 16 silent marker points in *gag* and *pol*, respectively.

### 2.2. Cell Culture

Human embryonic kidney (293T) cells were purchased from American Type Culture Collection and maintained in Dulbecco’s Modified Eagle’s Medium (DMEM) (Life Technologies, Grand Island, NY, USA) supplemented with 10% vol/vol heat inactivated cosmic calf serum (Hyclone, Logan, UT, USA) and 1% penicillin/streptomycin from Life Technologies.

Peripheral blood mononuclear cells (PBMCs) were isolated from buffy coats of HIV-1-seronegative blood donors (supplied by the Red Cross Blood Bank Service, Melbourne, Australia) by density gradient centrifugation over Ficoll-Plaque Plus (Amersham Biosciences, Uppsala, Sweden). The identities of the blood donors from Red Cross are anonymous. Peripheral blood lymphocytes (PBLs) and monocytes were purified from PBMCs by counter-current elutriation. Briefly, 5–10 × 10^8^ PBMCs were resuspended in 5 mL of FACS Wash (Dulbecco's phosphate-buffered saline (DPBS-), 1% fetal calf serum (FCS) and 2mM ethylenediaminetetraacetic acid (EDTA)). Cells were loaded into a standard elutriation chamber in a JE-5.0 rotor in a J-26 XP centrifuge (Beckman Coulter, Fullerton, CA, USA) at 600 × *g* cooled to 12 °C with a counter-centrifugal flow of FACS wash being pumped through the chamber at 12 mL/min. Smaller lymphocytes (T-cells, B-cells and natural killer (NK) cells) and residual platelets were collected into 50 mL conical tubes by allowing 1L of DPBS- to flow through the chamber and increasing the flow of the DPBS- to 15 mL/min. The flow of DPBS- was increased to 17 mL/min and monocyte enriched fractions were collected into 50 mL conical tubes. During collection the flow was increased by 1 mL/min every 100 mL until the monocytes were depleted from the chamber. Collection tubes were centrifuged at 320 × *g* for 10 min at 4 °C and monocytes were pooled. Elutriation of PBMC from buffy coats typically yielded 5–10 × 10^7^ monocytes. The purity of PBL and monocyte fractions was assessed by flow cytometry (FACSCalibur; Becton Dickinson, Franklin Lakes, NJ, USA) and estimated to be 90%–95% pure based on forward scatter (FSC) and side scatter (SSC) characteristics. PBLs were stimulated in RF10 media (2 × 10^6^ cells/mL) supplemented with 10 μg/mL polyhydroxyalkanoate (PHA) and 10 units/mL human interleukin-2 (IL-2) (Roche Applied Science, Sydney, NSW, Australia) for two days in Teflon-coated jars. PBLs were then resuspended in fresh medium containing 10 units/mL human IL-2 (Roche Applied Science) and incubated for a further two days before infection. Monocytes were cultured in IH10 medium, adherent to plastic, and allowed to differentiate into monocyte-derived macrophages (MDMs) for seven days before infection.

### 2.3. Virus Production

Viruses were produced by co-transfecting 293T-cells with HIV-1 molecular clones using polyethylenimine (PEI) [20]. PEI stocks were prepared at 1 mg/mL by dissolving PEI in water, adjusting the pH to 7.0, followed by filtration with a 0.2 μm sterile syringe filter. 2.5 × 10^6^ 293T-cells were seeded into 100 mm^2^ tissue culture plates 24 h prior to transfection. Transfection mix was prepared by adding 3 μg total HIV-1 proviral DNA to 500 μL of serum-free DMEM and 27 μL of PEI, vortexed and incubated for 5 min before addition to cells. 12 h post infection, cells were washed twice in DPBS- and the medium was replaced with fresh DMEM. Supernatants were collected 36 h post transfection and clarified by centrifugation for 30 min at 1462 × *g* at 4 °C to remove cellular debris. Clarified supernatant was then further purified by sequential filtration through 0.8 μm and 0.45 μm sterile syringe filter. Purified virus was then concentrated by ultracentrifugation through a 20% sucrose cushion using an L-90 ultracentrifuge (Beckman Coulter, Sudney, NSW, Australia) at 100,000 × *g* for 1 h at 4 °C. Pellets were resuspended in DMEM and virus quantified using the Vironostika HIV-1 p24 antigen enzyme-linked immunosorbant assay (ELISA) (BioMérieux, Marcy I’Etoile, France), according to the manufacturer’s instructions.

### 2.4. Infections

Concentrated viral stocks were supplemented with 2 mM MgCl_2_ and treated with 90 units/mL Benzonase (Sigma, Los Angeles, CA, USA) for 15 min at 37 °C to remove contaminating plasmid DNA before use. 2 × 10^6^ MDMs or stimulated PBLs were infected with 400 ng p24 equivalent of either homozygous or heterozygous virus, as determined by an HIV-1 antigen micro-ELISA. Cells were infected by spinoculation (1200 × *g* for 2 h at 25 °C). Six hours post-infection, 10 μg/mL T-20 (NIH AIDS Reagent Program) was added to the cells to prevent second-round replication. Cells were lysed in 200 μL PCR buffer (Roche) supplemented with 0.5% vol/vol Triton-X100, 0.5% vol/vol NP-40 and 150 µg/mL proteinase K, incubated at 56 °C for 1 h, and then 95 °C for 10 min. Cell lysates were stored at −20 °C and diluted 10 × with PCR-grade H_2_0 before quantification and amplification by quantitative PCR (qPCR). A number of control infections (described below) were also performed to control for potential experimentally-induced recombination and mutation events.

### 2.5. Quantitative PCR

HIV reverse transcription products were quantified using the HIV-1-specific primer pair M661/M667 [21], with copy number determined using ACH2 cell standards (containing a single integrated provirus) lysed and prepared as the experimental samples. To assess whether carryover plasmid from the transfection was successfully eliminated by the benzonase treatment (see Infections), we quantified plasmid DNA by targeting the ampicillin resistance gene with the primer pair Amp(s) AACTCGCCTTGATCGTTGGG and Amp(a) TGTTGCCATTGCTACAGGCATC. In all experimental samples, HIV-1 copy number due to plasmid carryover was less that 2% of that due to infection (average 1.37%, standard deviation 0.32%). In all experimental samples, HIV-1 copy number due to plasmid carryover was less that 2% of that due to infection (average 1.37%, standard deviation 0.32%). PCR conditions were 1× Brilliant II master mix (Stratagene, San Diego, CA, USA), 400 nM each primer, and 5 μL diluted cell lysate in a 15-μL reaction mixture volume. PCR conditions were an initial denaturation step at 95 °C for 15 min followed by 40 rounds of cycling at 95 °C for 10 s and then 60 °C for 30 s. Quantitative PCR was performed on an MX3000 instrument (Stratagene).

### 2.6. Amplifications for Sequencing

Pro-viral DNA was amplified from diluted cellular extracts using 14 sets of primers, generating 14 overlapping PCR amplicons. The primers are as follows: G1 sense (5′-GGTGCGAGAGCGTCGGTATTAAG-3′), G1 antisense (5′-CTGTGTCAGCTGCTGCTTGCTG-3′), G2 sense (5′-TCCTCTATTGTGTGCATCAAAGGATAGATG-3′), G2 antisense (5′-CCACTGTGTTTAGCATGGTATTTAAATCTTGTG-3′), G3 sense (5′-CAAATGGTACATCAGGCCATATCACCTAG-3′), G3 antisense (5′-CTGCATGCACTGGATGCAATCTATC-3′), G4 sense (5′-GAAGGAGCCACCCCACAAGATTTA-3′), G4 antisense (5′-GGTTCCTTTGGTCCTTGTCTTATGTCCAG-3′), G5 sense (5′-GGAAGTGACATAGCAGGAACTACTAG-3′), G5 antisense (5′-AGTCTTACAATCTGGGTTCGCATTTTGG-3′), G6 sense (5′-AAACTCTAAGAGCCGAGCAAGCTTC-3′), G6 antisense (5′-TGCCCTTCTTTGCCACAATTGAAACAC-3′), P1 sense (5′-GCAGGAGCCGATAGACAAGGAACT-3′), P1 antisense (5′-TAAAGTGCAGCCAATCTGAGTCAACAG-3′), P2 sense (5′-AGAAATCTGCGGACATAAAGCTATAGG-3′), P2 antisense (5′-GGAGTATTGTATGGATTTTCAGGCCCAA-3′), P3 sense (5′-GTAAAATTAAAGCCAGGAATGGATGGC-3′), P3 antisense (5′-GAAAAATATGCATCGCCCACATCCAG-3′), P4 sense (5′-TGTGGGCGATGCATATTTTTCAGT-3′), P4 antisense (5′-ATGGAGTTCATAACCCATCCAAAGGAATG-3′), P5 sense (5′-CACCAGCAATATTCCAGTGTAGCATG-3′), P5 antisense (5′-CTTTAATCCCTGCATAAATCTGACTTGCC-3′), P6 sense (5′-GAACTCCATCCTGATAAATGGACAGTACAG-3′), P6 antisense (5′-TTAAATGGCTCTTGATAAATTTGATATGTCCATTG-3′), P7 sense (5′-CCACTAACAGAAGAAGCAGAGCTAGAACTG-3′), P7 antisense (5′-CAGGTGGCTTGCCAATACTCTGTC-3′), P8 sense (5′-AGGGTGCCCACACTAATGATGTGAAAC-3′), P8 antisense (5′-AGTCTTCTGATTTGTTGTGTCCGTTAGG-3′). To avoid chimera formation due to over-amplification of cDNA during PCR, we checked that all primer pairs amplified with equal efficiency (Figure S1, Table S1 and [22]) and stopped the PCR in the log-linear phase, as previously described [22]. Each PCR well contained 2500 copies of template DNA, 1× HF buffer (Finnzymes, Sydney, NSW, Australia), 200 μM dNTP, 1 μM each primer, and 0.3 U of Phusion DNA polymerase (Finnzymes) in a 15-μL total reaction mixture volume. PCR cycling conditions were 98 °C for 30 s followed by 29 cycles of 98 °C for 10 s and 72 °C for 1 min. To reduce PCR bias, at least four independent PCRs were pooled for each amplicon. For each condition, replicates of the 14 different amplicons were pooled and unique six-nucleotide identifiers (barcodes) were attached using a modified parallel-tagged sequencing protocol to allow multiplexing on the same sequencing run [23]. Final 454 libraries for sequence were pooled from a total of 29 × 96-well plates of PCR (roughly 7 million initial DNA input templates). As the sequencing capacity was approximately 580,000 reads, not all of which were used due to alternative experimental setups and quality control, this ensured that resampling occurred at very low frequencies, if at all. Emulsion PCR and sequencing were performed at the Institute for Immunology and Infectious Diseases (IIID), Perth, Australia, according to standard GS FLX titanium procedures. A detailed discussion of the methodology, including comparisons of the different PCR amplification efficiencies, rationale for multiple PCR reactions, and detailed qPCR and amplification methods can be found in [16,22,24] and Figure S2.

### 2.7. Sequence Alignment

Sequences were aligned using Needle against a reference sequence. To reduce the error rate of 454 sequencing, sequences were removed if they were not full length or contained ambiguous nucleotides, as these reads are known to contain higher error rates [25]. All data were processed using custom software written in BioRuby [26]. Intervals that were incomplete or in which marker positions were mutated or ambiguous were discarded. Each interval was classified as recombined (if the markers at the endpoints switched between marker type and wild-type virus) or non-recombined (if marker endpoints were identical). Mutation frequencies were calculated for each interval.

### 2.8. Controls

**Figure 1:**
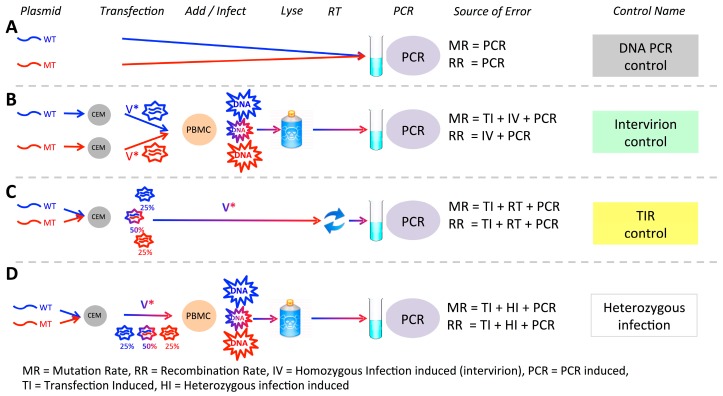
Experimental system and potential sources of error. Experimentally-induced recombination or mutation may arise at any point from plasmid production to sequencing. To control for this and to assess the contribution of different stages of processing to the observed rates, we analysed a variety of controls. All controls included underlying sequencing error, and in addition (**A**) DNA PCR control measures PCR induced recombination and mutation (**B**) Intervirion control measures PCR and homozygous infection induced recombination, and total mutation mutation rate due to transfection, infection and sequencing. (**C**) Transfection induced recombination control measures mutation and recombination rates due to transfection of CEM cells, reverse transcription of virus and PCR amplification. (**D**) Our experimental samples—heterozygous infection—allows us estimate the rate of infection-associated mutation and recombination.

Artificial mutation and recombination can be introduced during the experimental procedure and during sequencing. We have previously developed methods to estimate this background error rate and detailed them elsewhere [8,16,18], but briefly (i) to assess the background rate of mutation and recombination during PCR amplification and DNA sequencing, we PCR amplified a mixture of MK and WT plasmid DNAs (PCR control, Figure 1A, Table 1); (ii) to measure the rate of experimentally (PCR) induced recombination, as well the total mutation rate due to viral replication, co-infection of cells with multiple virions, library preparation and sequencing, we carried out two separate transfections of either the WT or MK plasmid DNA to produce homozygous WT and MK viruses. Equal amounts of these homozygous virus preparations were then used to infecT-cells, followed by cDNA extraction, amplification and sequencing (Intervirion control, Figure 1B, Table 1); (iii) to measure recombination and mutation potentially introduced during the experimental transfection step we extracted heterozygous RNA from 293T-cells and reverse transcribed it into cDNA by using SuperScript III (SSIII) (Invitrogen Life Technologies, Sydney, NSW, Australia) and gene-specific primer GAG4(4195)R (5’-ACATTTCCAACAGCCCTTTT TCCTAG-3’) to measure recombination and mutation potentially introduced during the experimental transfection step (TIR control, Figure 1C, calculated recombination rate = 0.005/1000nt). Since all recombination rates in controls were 3% or less than the recombination rate in heterozygous infection we were able to conclude that background recombination due to our experimental setup, does not bias our conclusions.

### 2.9. Mathematical Modeling

Since template switching is only observable in cells infected with heterozygous virus, and even then only when an odd number of switches occur between marker positions, we constructed a mathematical model to estimate the true template switching rate based only on the observed numbers of recombinations, while taking into account potential unobserved template switching events. This model has been previously described [8,18]. Briefly, the estimated recombination rate, *r*, can be calculated as

(1) $$r=\frac{\text{ln}\left(1-2\frac{n}{h}\right)}{2L}$$

where *L* is the length of the region over with recombination is being detected, *n* is the number of observed recombination events and *h* is the number of heterozygous sequences.

This model accounts for the possibility that multiple (and therefore unobserved) recombination events occurred over the interval. The number of heterozygous sequences is estimated directly from the data using the homozygous frequency of each virus type as described in [16]. The above method calculates the recombination rate as the number of recombination events per nucleotide per replication cycle (REPN). This measure is the basic measure of recombination and not dependent on sample size, or length between marker points. It can therefore be easily converted to other measures used in different systems such as multiplicity of infection for FACS based reporter protein experiments [16] and to the number of distinct recombinants produced for inter-subtype recombination.

### 2.10. Statistical Methods

Recombination rate calculations (and confidence intervals) were performed in R [27] using the generalized linear model function (glm) with a binomial error distribution. The recombination rate in each interval was calculated using Equation (1). This calculates an average recombination rate for each interval. To ensure the binomial error distribution, a custom link function identical to Equation (1) was used. Factors tested in the generalized linearized model (GLM) were cell type, viral phenotype, blood sample donor and interval region. Statistical significance of the covariates was tested using a chi-square test during an analysis of deviance. Correlations were performed in R using the function cor.test. Correlations are Pearson correlations unless otherwise stated. Power calculations were performed using G*Power 3.1 [28]. In our system we do not need to adjust for multiplicity of infection (MOI to calculate the recombination rate). This is because, unlike in FACs analysis of reporter proteins, our experimental setup allows for the direct sequencing of cDNA derived from individual infections which is unaffected by MOI.

### 2.11. Association between Mutation and Recombination

To test for an association between mutation and recombination we use our previously developed method that estimates the mutation rate per recombination event. Details of this method can be found in [8]. Briefly, we calculate both the number of mutations and the number of informative nucleotides occurring on intervals (i) with recombination (ii) without recombination from a sequence on which recombination was observed elsewhere (and so the intervals were derived from a heterozygous virion) and (iii) without recombination and from a sequence of unknown ancestry. We then optimise the expected value of the number of mutations in these three types of sequences using a model that incorporates the background mutation rate, the probability for observing a recombination and the rate of mutation per recombination.

## 3. Results

### 3.1. Experimental System

We have previously shown that 454 sequencing of reverse transcription products isolated from infected cells can simultaneously measure recombination and mutation [8,16,18]. Our experimental system is based on the insertion of silent “marker” codon modifications into *gag* and *pol* genes of the HIV genome. This allows recombination to be observed through the mixing of marker points between co-packaged WT and MK genomes. Furthermore, each marker point consists of at least two mutations in consecutive codons allowing recombination and mutation to easily distinguished.

To simultaneously measure the recombination and mutation rate due to viral replication, we produced heterozygous virus particles by co-transfection of WT and MK plasmids into 293T-cells, resulting in a mixture containing 50% heterozygous virions, 25% homozygous WT virions, and 25% homozygous MK virions (Figure 1D). Using the same batch of virus, we then carried out parallel infections of T-cells (peripheral blood lymphocytes isolated from buffy coats) and macrophages (monocyte-derived macrophages) from three matched blood donors. Importantly, we were able to estimate the proportion of heterozygous virions used to infect both T-cells and macrophages from the data and found this to be, as expected, almost exactly 50% (range 49.85%–49.97% in T-cells and 49.95%–50.00% in macrophages). Finally, we have previously shown that the background levels of recombination and mutation due to sequencing errors and sample preparation to be low in our experimental setup (Table 1) [8].

### 3.2. Higher Recombination Rate in Macrophages Compared to T-cells

Two studies have observed higher rates of recombination in macrophages compared to T-cells [14,15], but this has not been universally observed [13]. We therefore carried out infection of both T-cells and macrophages with HIV-1 virus from the blood of three matched donors. We used virus expressing the C-C chemokine receptor type 5 (CCR5)-tropic AD8 envelope, as these viruses can infect both T-cells and macrophages [29]. Of note, this allowed us to use the same batch of virus for each experiment, so that we could exclude batch-to-batch variations in virus preparation as an explanation for any differences observed.

**Figure 2:**
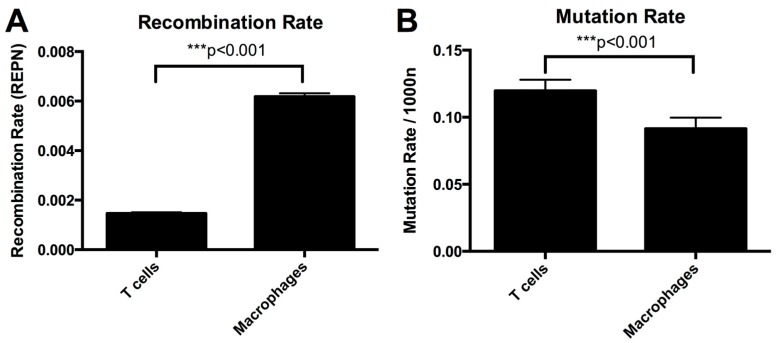
Recombination and mutation rates in T-cells and macrophages. Both panels show estimated rates and 95% confidence intervals.

The average recombination rate in T lymphocytes was 1.46 × 10^−3^ recombination events per nucleotide per round of infection (REPN) (95% CI 1.42–1.51 × 10^−3^ REPN) and in macrophages it was 6.18 × 10^−3^ REPN (95% CI 6.05–6.32 × 10^−3^ REPN) (Table 2 and Figure 2A). The recombination rate was significantly higher in macrophages than in T lymphocytes (*p* < 0.001, GLM model). A higher recombination rate in macrophages than in T-cells was observed in all three donors (Table 3).

### 3.3. The Difference in Recombination Rate between T-cells and Macrophages Is Not due to Means of Co-Receptor Entry or Sub-Population of T-cells

HIV can enter cells through either the CCR5 or the on C-X-C chemokine receptor type 4 (CXCR4) co-receptor. Both memory T-cells and macrophages can be infected via the CCR5 co-receptor, while naïve T-cells are infected via the CXCR4 co-receptor. We therefore sought to determine whether the means of viral entry in T-cells affects the recombination rate. We repeated our work using virus expressing the NL4-3 envelope, which enters through the CXCR4 co-receptor. Thus, it can infect naïve T-cells as well as memory T-cells. We used our GLM model to estimate the recombination rate in 5 matched donors infected with CCR5-tropic and CXCR4-tropic virus. We found the contribution to the recombination rate of the different virus types to be between 5%–10% of the total recombination rate. This contribution was less than the variation observed in infection between different blood donors. Additionally, it was an order of magnitude smaller than the difference in recombination rates between T-cells and macrophages (95% CI on difference in recombination rate between virus types is 5.35 × 10^−5^–1.61 × 10^−4^ REPN). Thus, the difference in recombination rates observed between macrophages and T-cells does not appear to be due to the means of co-receptor entry, co-receptor signaling, or the sub-population of T-cells considered.

### 3.4. Hot and Cold Spots of Recombination Are Similar between T-cells and Macrophages

**Figure 3:**
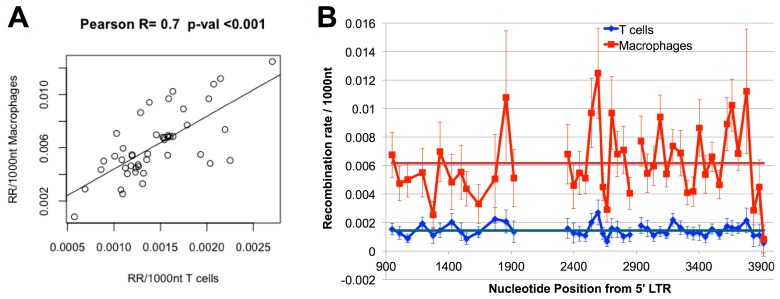
Recombination rates in T-cells and macrophages by interval. (**A**) Recombination rate in each interval in macrophages is correlated with the recombination rate in the same interval in T-cells (*r* = 0.7, *p* < 0.001) Note the different scales used on the x and y axes. (**B**) Recombination rates in macrophages and T-cells in each interval. 95% confidence intervals are shown (after Bonferroni correction for multiple comparisons). Dark lines indicate the average recombination rate (RR) across all intervals for T-cells (blue) and macrophages (red).

The increased recombination rate observed in macrophages could be either due to an increase in recombination rate across the entire genome, or due to localised differences between the cell types. We have previously identified regions of the HIV genome that are hot and cold spots for recombination in T-cells [18] and it is possible that one or more recombination hotspots that are present only in macrophages (or cold spots that are present only in T-cells) are driving the observed differences. To differentiate between the above two hypotheses, we compared the recombination rates in both T-cells and macrophages across segments of *gag* and *pol*. We found that the recombination rate was higher in macrophages than in T-cells in every interval examined, and that in 47/48 intervals this difference was significant at a level of *p* = 0.01. Additionally, we found a high and significant correlation between the recombination rates in matched intervals between T-cells and macrophages (*r* = 0.72, *p* < 0.001, Pearson correlation). As we have previously observed that the marker system itself does not influence recombination rate [18], taken together these analyses indicate that recombination hot and cold spots are similar in the two cell types (Figure 3) and the increased recombination rate in macrophages is a genome-wide effect.

### 3.5. Mutation Rate in Macrophages Is Lower than in T-cells

**Figure 4:**
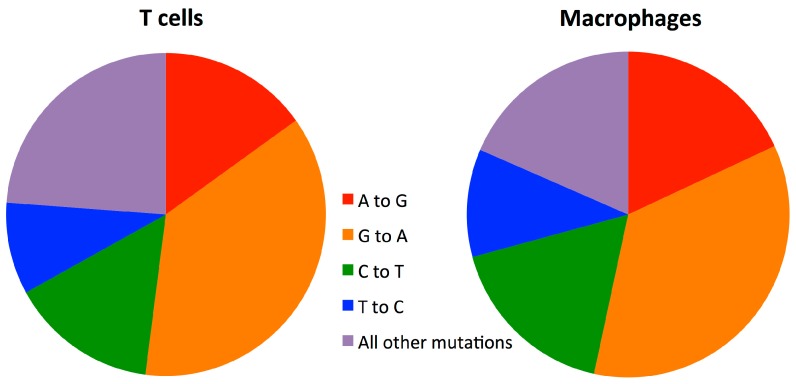
Pie charts showing patterns of mutations in T-cells and macrophages. Specific transitions are shown as they represent the highest rates of mutation.

We next compared the substitution mutation rates in macrophages and T-cells. We found the mutation rate in macrophages to be 0.09/1000nt and in T-cells to be 0.12/1000nt (Table 2 and Figure 2B). Macrophages had a significantly lower mutation rate than T-cells (*p* < 0.001, Fisher’s Exact Test). A lower mutation rate was found in macrophages compared to T-cells in all three donors (Table S1). Importantly, we found no clear differences in the mutation patterns observed in T-cells and macrophages (Table 4 and Figure 4). This equality in the type of mutation also provides evidence that the difference between macrophages and T-cells is not a product of unequal nucleotide sample sizes (Table 4) arising from selection of sequences based on sequencing quality (see sequence alignment in Methods).

We next tested whether we could find an association between mutation and recombination in macrophages. We have previously shown that in T-cells, sections of the genome that have undergone recombination have a significantly higher mutation rate than sections that have not recombined [8]. In contrast, here we found that there was no significant difference in mutation rate between recombined and non-recombined sections in macrophages (mutation rate 0.084/1000nt on recombined intervals *versus* 0.092/1000nt on non-recombined intervals; *p =* 0.56 by Fisher’s exact test). We also tested for an association between mutation and recombination in macrophages using a mathematical model that accounts for multiple unobserved recombinations that we previously developed for this purpose [8]. Once again, and in contrast to T cells, our test did not uncover any association between recombination and mutation in macrophages. Finally, to investigate whether this lack of association was due to insufficient statistical power, we performed a power calculation and found that if the recombined *versus* non-recombined intervals in macrophages had the same relative difference in mutation rates in that was previously observed in T-cells [8], we would have a 96% power to detect a difference in mutation rates at 5% significance. Additionally, we found that at the 95% confidence limit, recombined regions could have a higher mutation rate than non-recombined intervals by at most a 0.016/1000nt, only a quarter of the difference previously observed in T-cells [8].

## 4. Discussion

By directly measuring the template switching rate in HIV genomes we have shown that recombination occurs at a significantly higher rate in macrophages than in T-cells. This increase in recombination is not due to the specific localised hotspots of recombination present in macrophages, as the recombination rates were higher across the Gag and Pol genes in macrophages. The lower recombination rate in T-cells still occurred when T-cells were infected via the CXCR4 co-receptor, although the difference in recombination rates in cells infected via either the R5 or X4 co-receptors is unlikely to be biologically significant. We found no clear differences in the patterns of mutations observed in T-cells and macrophages, indicating that the classes of mutations occurring are unlikely to be responsible for the different observed mutation rates, in contrast to what was observed as being the difference in mutation rates between HIV-1 and HIV-2 [30].

We have previously shown an association between recombination and mutation in T-cells, suggesting that 15%–20% of the mutations observed in CD4+ T-cells occur in association with recombination events [8]. Thus, we expected that the higher recombination rate in macrophages may drive a higher mutation rate. Contrary to our expectations, the increased recombination rate observed in macrophages was not associated with an increase in mutation rate. Moreover, we observed no such association between recombination and mutation in macrophages. This suggests that different dynamics may be governing the reverse transcription in the two cell types.

**Figure 5:**
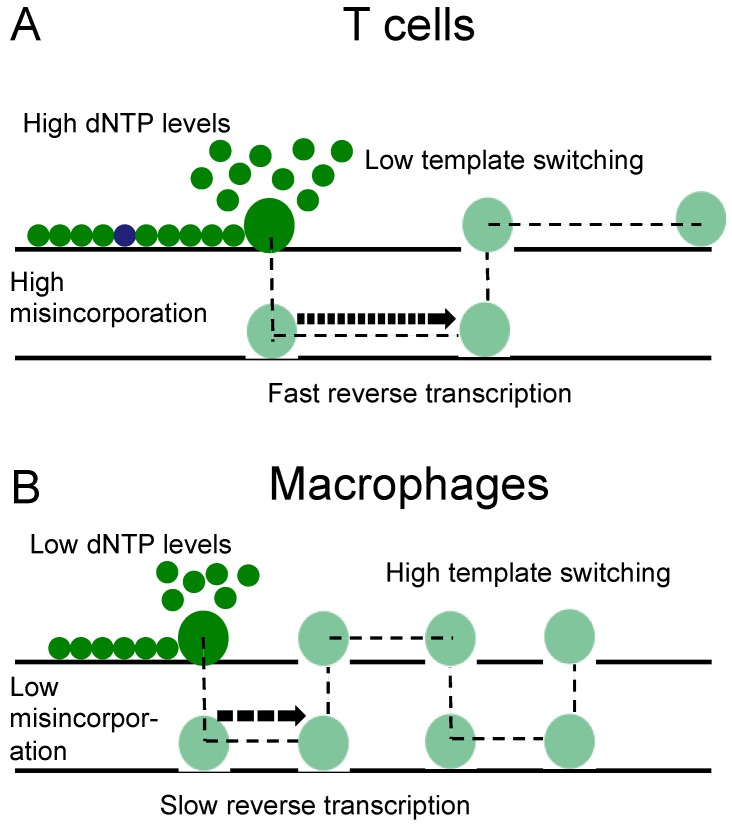
Reverse transcription associated with different dNTP concentrations in T lymphocytes and macrophages. (**A**) T-cells have high dNTP concentrations and associated with this higher mutation rates but lower rates of strand transfer; (**B**) monocyte-derived macrophages (MDMs) have lower dNTP concentrations and associated higher rates of strand transfer, but lower mutation rates.

It has been shown that lower concentrations of dNTPs, at the level that is seen in macrophages, can introduce pausing and delay DNA synthesis [31]. Others have shown that the interaction between HIV reverse transcriptase (RT) and dNTP substrate can contribute to viral mutagenesis *in vitro*, in particular within the context of HIV RT mutants (V148I and Q151N) that are defective in dNTP bindings [32]. In addition, at lower dNTP concentrations HIV shows higher strand transfer efficiency [33] and increased template switching [34], leading to the conclusion that changes in cellular dNTP levels may alter the rate of HIV template switching [15]. These observations are consistent with the results presented here, that macrophages have both a higher template switching rate and a lower mutation rate than T lymphocytes. We therefore hypothesise that the higher rate of template switching and associated lower rate of mutation in macrophages may be a direct result of the lower levels of dNTPs in these cells (Figure 5). This model is consistent with the observation that high levels of dNTP hydrolyzing enzyme SAM domain and HD domain-containing protein 1 (SAMHD1) in macrophages are associated with a higher rate of HIV recombination [15].

Establishing mutation and recombination rates in the primary targeT-cells of HIV is essential for understanding immune escape, drug resistance and viral evolution. We have shown that both the mutation rate and recombination rate of HIV are different between T-cells and macrophages, and that the conditions governing mutation and recombination in these cell types have led to distinct outcomes that may impact on virus evolution.

## Figures and Tables

**Table 1:** Control results from experimental infections.

Control Type	# Nucleotides	MR/1000nt	RR/1000nt *
**PCR**	4,931,024	0.074	0.015
**Intervirion (T-cells)**	15,407,933	0.119	0.044
**Intervirion (macrophages)**	10,947,705	0.097	0.047

Very little recombination is observed either due to PCR amplification and DNA sequencing or homozygous infection. * note that the recombination rate is not simply the number of recombination events divided by the number of nucleotides, but uses the method described in [16] that takes into account the potential for unobserved recombinations to have occurred. MR: mutation rate; RR: recombination rate

**Table 2:** Results from experimental infections.

Cell Type	# Nucleotides	# Recombs	# Mutations	MR/1000nt	RR/1000nt *
**T-cells**	7,180,718	4801	859	0.120	1.465
**Macrophages**	5,731,387	12,769	524	0.091	6.184

* note that the recombination rate is not simply the number of recombination events divided by the number of nucleotides, but uses the method described in [16] that takes into account the potential for unobserved recombinations to have occurred.

**Table 3:** Results from experimental infections split by donor.

Donor	RR/1000nt T-cells	RR/1000nt Macrophages	MR/1000nt T-cells	MR/1000nt Macrophages
**Donor A**	1.73	3.82	0.120	0.092
**Donor B**	1.13	7.25	0.118	0.085
**Donor C**	1.55	7.61	0.121	0.098

Macrophages show a higher recombination rate and lower mutation rate than T-cells in all three donors.

**Table 4:** Mutation patterns in T-cells and macrophages.

	T-cells						Macrophages					
Orig Nucl	# Nucl	% of Muts	% Mutating to				# Nucl	% of Muts	% Mutating to			
			A	C	G	T			A	C	G	T
**A**	2,745,580	20.6	0.0	3.0	15.1	2.4	2,275,211	22.2	0.0	1.1	18.1	3.0
**C**	1,300,503	22.8	6.8	0.0	1.2	14.9	1,069,497	24.2	5.5	0.0	1.3	17.4
**G**	1,576,904	42.4	36.9	1.0	0.0	4.4	1,297,412	40.5	35.3	1.1	0.0	4.1
**T**	1,557,725	14.2	2.2	9.2	2.8	0.0	1,293,501	13.1	0.2	10.7	2.2	0.0

## Supplementary Files

Supplementary File 1
