# Supplementary material for: HIV-1 Mutation and Recombination Rates Are Different in Macrophages and T-cells
Source: Viruses. 2016 Apr 22;8(4):118. doi: 10.3390/v8040118 (PMC4848610; doi:10.3390/v8040118)
Supplement: Supplementary File 1 [file viruses-08-00118-s001.docx]

Supplementary Material: HIV-1 Mutation and Recombination Rates Are Different in Macrophages and T-cells

Deborah Cromer, Timothy E. Schlub, Redmond P. Smyth, Andrew J. Grimm, Abha Chopra, Simon Mallal, Miles P. Davenport and Johnson Mak


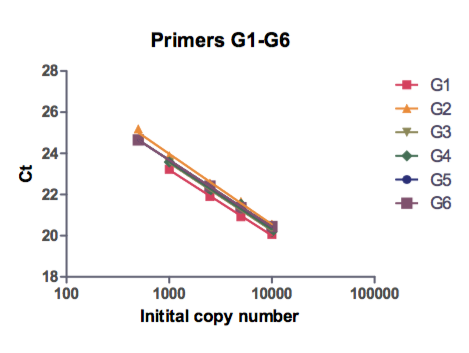


**Figure S1.** Linearity of PCR for primer pairs G1-6.

**Table S1.** Efficiency of primer pairs G1-4 and P1-6.

| **Primer** | **R^2^** | **Slope** | **Efficiency** |
| --- | --- | --- | --- |
| G1 | 0.999 | −3.19 | 105.91 |
| G2 | 0.956 | −3.43 | 95.72 |
| G3 | 0.999 | −3.29 | 101.44 |
| G4 | 0.987 | −3.28 | 101.82 |
| G5 | 1.000 | −3.25 | 103.18 |
| G6 | 1.000 | −3.25 | 103.18 |


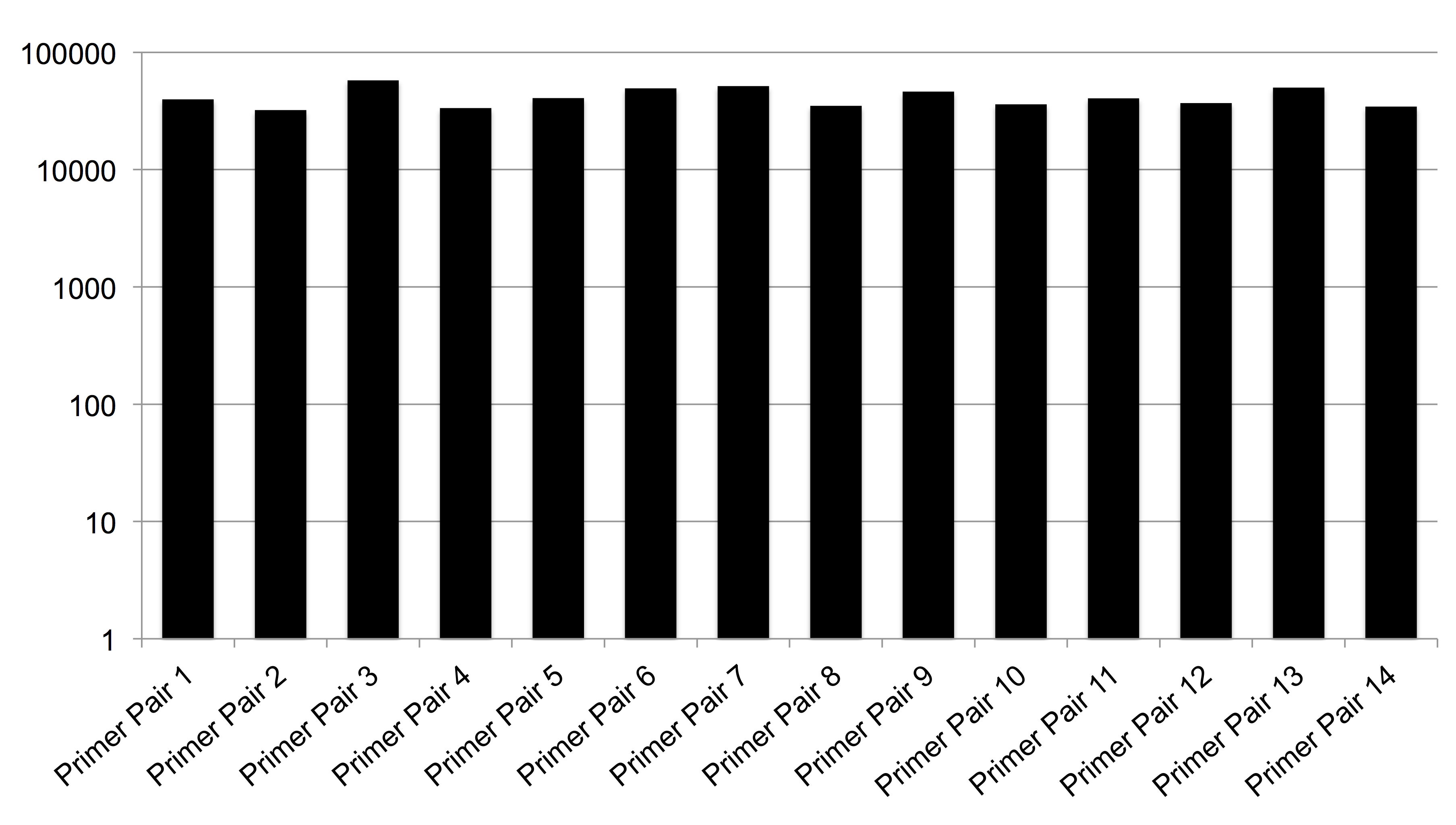


**Figure S2.** Bar chart of sequences obtained for each primer pair.
